# Problems of three high focus Northeastern states after five years of decentralised planning in India

**DOI:** 10.1186/1753-6561-6-S1-P10

**Published:** 2012-01-16

**Authors:** Alivia Biswas, Sweta Roy

**Affiliations:** 1 MedicaSynergie Private Limited, India

## Introduction

National Rural Health Mission (NRHM) was launched to address with a mission approach all components of functional systems; infrastructure, human resources, logistics and participation of the community through increasing government investment in healthcare and addressing managerial weaknesses in the system.

Before the launch of NRHM, in order to bring the states that were performing poorly in terms of health indicators at par with the better performing states, the empowered action group of eight states was constituted in 2001. The failure to achieve desirable improvement in health indicators led the NRHM to focus on 18 poorly performing states, which included eight northeastern states of Assam, Meghalaya, Tripura, Manipur, Nagaland, Mizoram, Arunachal Pradesh and Sikkim.

Our consultancy organisation got an opportunity to be part of the decentralised planning process in three high focus northeastern states of Meghalaya (2006-2007), Tripura (2006-2007) and Sikkim (2007-2008) under NRHM. After five year's journey of NRHM, this study aims at revisiting the situation in the light of the performance of these three states in three identified components of structural correction of the health system.

## Methods

We collected primary information through in-depth interviews of key functionaries. We also did a desk review of secondary literature.

## Results

Shortage of human resource in the three states is a major problem in health sector in explaining ineffective and inefficient health outputs. Tripura is yet to appoint Auxiliary Nurse Midwives (ANM) in about 8.5% of health sub-centres in order to make them functional. The placement of a proposed second ANM at health sub-centre is a mammoth challenge. All the primary health centres (PHC) in Sikkim and 13% of PHCs in Meghalaya do not have staff nurse. In Meghalaya 13% of its PHCs offer services round the clock.

The presence of the medical care is concentrated in and around semi-urban and urban conglomerates as the overall development issues are yet to be addressed for the hilly areas. The availability of clinical cadre is often in papers. The demand by Accredited Social Health Activists (ASHA), recently introduced female health workers at village levels, for being included in the mainstream is adding to the human resource issues to be resolved. It has remained a challenge to find clinical specialists for placements at community health centres (referral facilities).

*Janani Suraksha Yojana* (Maternity protection scheme) has enabled Meghalaya to achieve 10% increase in institutional deliveries, the highest among the three states, but failed in assuring 48 hours of recommended stay of women in PHCs mainly due to unavailability of manpower, lack of electricity, and related amenities. Political unrest and inaccessible geographic terrains of three states often add to the multitude of the problem.

Development of Indian Public Health Standards (IPHS) under NRHM has been an important effort towards assuring quality healthcare services. However none of the healthcare facilities in the three states have achieved these standards.

In regard to flexible financing under NRHM all the three states exhibited gradual increase in expenditure pattern till the year 2008-2009 with a fall in the year 2009-2010. The gradual increase in financial allocation and disbursements with reduction in expenditure implies inability of the states to spend the available fund. The utilisation of untied fund available with health sub-centres and village level committees increased from the year 2005 to 2010. However they exhibited inability to submit utilisation certificates for finances expended.

## Discussion

If we remove the performance indicators pertaining to Assam from the northeastern states, the non-performance of other northeastern states becomes apparent. Also the financial allocation is skewed if Assam is seen in along with other northeastern states. The uniqueness and variety of northeastern states cannot fall into the “one size fit all” interventions and strategies. For example, appointment of ASHAs has led to resentment among trained birth assistants supporting home deliveries in hilly terrain and led to confusion in a successful service delivery mechanism.

We feel that the norms relating to two ANMs at health sub-centres and having healthcare facilities functioning round the clock can be relaxed and more attention need to be given on integration of local and other healing traditions into present healthcare services. NRHM has provided an impetus to utilisation of healthcare services but lack of concurrent development has affected the impression of the user negatively.

